# Comparative Effectiveness of Carbetocin Versus Oxytocin in Active Management of the Third Stage of Labour for Preventing Atonic Postpartum Haemorrhage: A Study at a Tertiary Care Centre in Central India

**DOI:** 10.7759/cureus.93537

**Published:** 2025-09-30

**Authors:** Nalini Mishra, Prajesh Darji, Aditi Singh, Nishi Mitra, Devanshi Mishra

**Affiliations:** 1 Obstetrics and Gynaecology, Lakshmi Narain (LN) Medical College and Research Center, Bhopal, IND; 2 Obstetrics and Gynaecology, Pacific Institute of Medical Sciences, Udaipur, IND; 3 Obstetrics and Gynaecology, Bansal Hospital, Bhopal, IND

**Keywords:** active management of the third stage of labour, heat-stable carbetocin, mean blood loss, oxytocin, postpartum haemorrhage (pph)

## Abstract

Introduction: We aim to determine the comparative effectiveness of heat-stable carbetocin (HSC) versus oxytocin for the prevention of atonic postpartum haemorrhage (PPH) at a tertiary care centre in Central India.

Method: This study is a prospective observational study conducted over 18 months (January 2023 to July 2024) and included 284 low-risk gravid women in labour at term after obtaining ethical approval and informed consent. There were 142 participants in each group. Group 1 received heat-stable carbetocin 100 mcg, and group 2 received oxytocin 10 units intramuscularly within one minute of delivery. Women with medical or obstetrical disorders were excluded. The primary objective was to compare the incidence of atonic PPH among participants receiving carbetocin versus oxytocin after the delivery of the baby. The secondary objective was to estimate the mean blood loss (MBL) and change in the haemoglobin (Hb) level before and after delivery in the two groups. Blood loss was estimated by quantifying blood loss by adding up the volumetric component (using a calibrated drape or suction bottle) to the gravimetric estimate. The incidence of PPH and the mean blood loss were noted. The degree of haemoglobin decline before and after delivery was also recorded. Statistical significance was assessed using Student’s t-test and chi-square test, and a p-value of <0.05 was taken as significant.

Result: The incidence of PPH was comparatively less in women in the carbetocin group (group 1) when compared with those in the oxytocin group (group 2), although not statistically significant (4.2% versus 7%, respectively; p=0.303). A significant difference was observed in the mean blood loss, being less with carbetocin (623±81.3 versus 678±88.7 mL; p=0.039). Haemoglobin decline was also found to be less in those receiving carbetocin (p<0.001).

Conclusion: Although insignificant statistically, carbetocin plays an effective role in preventing PPH when compared to oxytocin, particularly in settings where cold chain maintenance for oxytocin transport and storage remains doubtful. Statistically significant lower mean blood loss with carbetocin can be an added advantage.

## Introduction

Postpartum haemorrhage (PPH) complicates approximately 1%-10% of all deliveries and is the leading cause of maternal death. It currently accounts for approximately 25% of pregnancy-associated deaths worldwide [1]. Improvement in PPH survival begins with holistic strengthening of each step along the continuum of care in health systems [2]. The cause of PPH is atonicity of the uterus in 70%-80% of instances. Preventing PPH caused by uterine atony plays a significant role in improving maternal mortality and morbidity. The use of uterotonic agents as the most important component of active management of the third stage of labour (AMTSL) has been shown to reduce the rate of PPH by preventing atonicity of the uterus in significant proportions [3].

The World Health Organization (WHO) recommendation for pharmacological means of PPH prevention advocates the use of oxytocin (10 IU intramuscularly (IM) or intravenously (IV)) and carbetocin (if cost-effective), ergot alkaloids (alone or combined if there are no contraindications) or oral misoprostol in settings where oxytocin is not available or its quality cannot be guaranteed [4]. The most commonly used agent at present is oxytocin owing to its affordable cost and wider availability. The onset of action when IM oxytocin is given is within 3-7 minutes; however, it has the drawback of a short half‑life of 1-6 minutes, which often necessitates further continuous intravenous infusion or multiple intramuscular injections. Another important concern with oxytocin is the need to maintain the cold chain of temperature ranging between 2°C and 8°C in order to retain its full potency [5]. This is very difficult in low- and middle-income countries (LMICs), and failure to do so results in suboptimal quality of the drug. A high prevalence of poor-quality oxytocin samples having inadequate amounts of active ingredient has been reported in LMICs due to poor storage and transportation [6-8].

Carbetocin is a long-acting synthetic analogue of oxytocin. It has a quick start (within 1-2 minutes) and a long duration of action (around one hour). It acts by coupling with oxytocin receptors on the uterus, which leads to rhythmic contractions and enhances the rate and strength of existing contractions, resulting in enhanced uterine tone. Its safety profile is similar to that of oxytocin [9]. Recently, the effectiveness of carbetocin has been evaluated in low- and middle-income countries because, unlike oxytocin, it can be used at ambient temperatures and thus may have the potential to be more effective in preventing PPH. The Carbetocin Haemorrhage Prevention (CHAMPION) trial showed that heat-stable carbetocin (HSC) was no different from oxytocin for the prevention of blood loss of at least 500 mL or the use of additional uterotonic agents [10]. Heat-stable carbetocin is a uterotonic recommended only for PPH prevention and was added by the World Health Organization (WHO) to the core list of reproductive health medicines in the 2019 Model List of Essential Medicines [11].

Thus, the present study was conducted with the objective of determining the comparative effectiveness of heat-stable carbetocin (HSC) versus oxytocin for the prevention of atonic postpartum haemorrhage when administered immediately within one minute after the delivery of the baby at a tertiary care centre in the central part of India.

## Materials and methods

The present study was conducted to determine the comparative effectiveness of heat-stable carbetocin (HSC) versus oxytocin for the prevention of atonic postpartum haemorrhage when administered immediately within one minute after the delivery of the baby at a tertiary care centre in the central part of India.

Primary outcome

The primary objective was to compare the incidence of primary atonic postpartum haemorrhage among participants receiving carbetocin versus oxytocin after the delivery of the baby.

Secondary outcome

The secondary objective was to estimate the mean blood loss (MBL) and change in the haemoglobin (Hb) level before and after delivery in the two groups.

Study design

The present study is a single-centre, prospective observational study conducted from January 2023 to July 2024 after obtaining approval from the Institutional Ethics Committee of LN Medical College and Research Centre and JK Hospital (reference number: LNMC & RC/Dean/2022/Ethics/054 dated 24/12/22).

The study included 284 women (142 in each group) selected through convenience purposive sampling. Informed consents were taken from all participants. Group 1 received carbetocin 100 mcg intramuscularly (IM), whereas group 2 received oxytocin 10 IU IM within one minute of delivery. Sample size was calculated using Slovin’s formula: n = N/(1+Ne2), where N is the population size and e is the margin of error.

Inclusion criteria

We included women with full-term (>37 weeks of gestation), singleton pregnancy, who are low risk (without any medical or obstetric high-risk factors) and expecting vaginal or caesarean section delivery, and who gave consent for the study.

Exclusion criteria

Women with medical disorders of pregnancy, such as hypertension (hypertensive disorders of pregnancy), cardiac, renal and liver disease, epilepsy and severe anaemia; those with obstetric high-risk factors such as polyhydramnios, multiple gestation, antepartum haemorrhage and morbid adhesion of placenta; those on anticoagulant therapy; and those with bleeding disorder or any coagulopathy were excluded from the study. Those cases of PPH where the cause was found to be other than atony of the uterus were also excluded.

A detailed history and a thorough clinical examination of every woman were completed by the obstetric team. Appropriate laboratory investigations (particularly haemoglobin level before delivery) were recorded. The labour events were recorded in detail. The participants were given either oxytocin or carbetocin at the discretion of the treating obstetric unit. There were a total of 142 women in group 1 who received prophylactic heat-stable carbetocin (HSC) 100 mcg intramuscularly for the prevention of PPH immediately after delivery of the baby (within one minute), and the same number of women (142) received oxytocin 10 IU in group 2. According to the latest guidelines on PPH prevention by the Federation of Obstetric and Gynecological Societies of India (FOGSI), also advocated by the International Federation of Gynecology and Obstetrics (FIGO) and WHO, oxytocin (10 IU IV/IM) should be given within one minute of the delivery of the baby after excluding the second twin. So, both oxytocin and carbetocin were given within one minute of delivery [12].

Blood loss was estimated in each woman of both groups by quantification of blood loss (QBL), which was derived by adding up the volumetric component (using a calibrated drape or suction bottle, after excluding the amount of liquor amnii) to the gravimetric estimate (difference of weight between soaked and dry mops and sponges and considering each gram of difference as 1 mL of blood loss). Women were observed for two hours after delivery. During these two hours, uterine tone was assessed, and vital signs were recorded every 15 minutes. After this initial monitoring, the patients were transferred to the postpartum ward.

The incidence of PPH, defined as a bleed of 500 mL or more in vaginal deliveries and in excess of 1000 mL in caesarean deliveries, was noted in the two groups [13]. The participants were simultaneously assessed for the side effects of the drugs, such as headache, hypotension and vomiting not attributable to other obstetric causes. In case of PPH, it was managed as per protocol until haemostasis. The need for additional uterotonics to arrest bleeding was noted; other interventions were also recorded, such as the number of blood transfusions, the need for second-line interventions or surgical treatment for refractory PPH and the need for intensive care unit (ICU) admissions.

Repeat Hb test was done after 24 hours of delivery. The occurrence of complications was monitored until the participants were discharged from the hospital.

Statistical analysis

The collected data were entered into SPSS version 20 (IBM Corp., Armonk, NY) for analysis. Quantitative data confirming the properties of the normal distribution are presented as means ± standard deviation (SD). For discrete data, the author calculated and reported frequency, proportion and percentage. Statistical significance for the differences between the two groups was assessed using Student’s t-test and chi-square test. A p-value of <0.05 was taken as significant.

## Results

The epidemiological and obstetric characteristics of the women included in the study were comparable, with no statistically significant variation. The mean age in both study groups was 27 years. The majority of the women were multiparous (≥1 previous birth) in both groups. The mean gestational age at delivery or caesarean section was around 39 weeks. Body mass index (BMI) was also similar in both groups. Women with pre-labour moderate anaemia were slightly more in the carbetocin group (Table 1).

**Table 1:** Demographic and epidemiological characteristics of the study participants in both groups Group 1: carbetocin, group 2: oxytocin Mild anaemia: Hb = 10-10.9 g/dL, moderate anaemia: Hb = 7-9.9 g/dL Hb: haemoglobin, SD: standard deviation

Parameter	Group 1 (n=142) (number (%))	Group 2 (n=142) (number (%))	p-value
Age (years) (mean±SD)	27.3±4.05	27.1±4.09	0.732
Parity			
Nulliparous	50 (35.2)	45 (31.7)	0.34
Multiparous	92 (64.8)	97 (68.3)	0.4
Gestational age (weeks) (mean±SD)	39.3±1.3	38.6±1.5	0.0893
Body mass index (kg/m^2^)	23.3±1.9	22.9±2.1	0.372
Anaemia (pre-labour)			
Mild	61 (43)	58 (40.8)	0.089
Moderate	23 (16.2)	16 (11.3)	0.094

The number of vaginal deliveries and caesarean sections were almost similar in both carbetocin and oxytocin groups. It was seen that 44 (54.32%) and 41 (56.16%) of labour were spontaneous in onset in both groups. Thirty-five (43.2%) vaginal deliveries in the carbetocin group had an episiotomy, whereas this number was slightly higher in the oxytocin group (38 (52.1%)) (Table 2).

**Table 2:** Labour and delivery characteristics of the study participants in both carbetocin and oxytocin groups (n=142) Group 1: carbetocin, group 2: oxytocin

Characteristics	Group 1 (number (%))	Group 2 (number (%))	p-value
Mode of delivery			
Vaginal	81 (57)	73 (51.5)	0.0832
Caesarean section	61 (43)	69 (48.5)	0.0734
Onset of labour			
Spontaneous	44 (54.32)	41 (56.16)	0.721
Induced	13 (16.05)	14 (19.18)	0.431
Augmented	20 (24.69)	18 (24.66)	0.603
Episiotomy			
No	46 (56.8)	35 (47.9)	0.431
Yes	35 (43.2)	38 (52.1)	0.284

The incidence of PPH was six (4.2%) in the carbetocin group and 10 (7%) in the oxytocin group, which is not statistically significant. However, there was a significant difference observed in the mean blood loss (MBL) in the two groups, with an overall amount of 623±81.3 and 678±88.7 mL, respectively, in the carbetocin and oxytocin groups (p=0.039). The majority of PPH cases were managed with additional uterotonic agents. One case from each group required pelvic devascularisation, and no obstetric hysterectomy was performed. Two (1.4%) cases required blood transfusion in the carbetocin group, whereas three (2%) cases required blood transfusion in the oxytocin group (Table 3).

**Table 3:** Incidence of PPH and PPH management strategies in both groups (n=142) Group 1: carbetocin, group 2: oxytocin PPH: postpartum haemorrhage, SD: standard deviation, OBS: obstetric

Characteristics	Group 1 (number (%))	Group 2 (number (%))	p-value
Postpartum haemorrhage			
No	136 (95.8)	132 (93)	0.303
Yes	6 (4.2)	10 (7)	
Mean blood loss (mean±SD)	623±81.3	678±88.7	0.039
Postpartum haemorrhage treatment			
Managed with medical methods	3	5	-
Needed balloon tamponade	1	2	-
Needed compression suture	1	2	-
Needed devascularisation	1	1	-
Needed OBS hysterectomy	0	0	-
Need for blood transfusion			
No	140 (98.6)	139 (98)	0.183
Yes	2 (1.4)	3 (2)	

When the vaginal mode of delivery was taken into consideration, the incidence of PPH was higher in group 2 (oxytocin), which was nine (12.33%), as compared to group 1 (carbetocin), which was four (4.9%). However, this was not statistically significant. It was almost similar when caesarean sections were considered (Table 4).

**Table 4:** Incidence of PPH according to the mode of delivery Group 1: carbetocin, group 2: oxytocin PPH: postpartum haemorrhage

PPH	Vaginal delivery in group 1 (n=81) (number (%))	Vaginal delivery in group 2 (n=73) (number (%))	p-value
Yes	4 (4.9)	9 (12.33)	0.1
No	77 (95.1)	64 (87.67)	
PPH	Caesarean delivery in group 1 (n=61) (number (%))	Caesarean delivery in group 2 (n=69) (number (%))	p-value
Yes	2 (3.2)	1 (1.5)	0.488
No	59 (96.8)	68 (98.6)	

The mean blood loss also varied according to the mode of delivery, being significantly less in group 1 in comparison to group 2 when caesarean sections were considered (733 ±109.8 versus 798±128.7; p≤0.0001), whereas this difference in the two groups was less in those delivered vaginally (Table 5).

**Table 5:** Mean blood loss in both groups according to the mode of delivery Group 1: carbetocin, group 2: oxytocin MBL: mean blood loss

Parameter	Vaginal delivery in group 1 (n=81) (number (%))	Vaginal delivery in group 2 (n=73) (number (%))	p-value
MBL (mL)	373±79.8	394±89.7	0.03
Parameter	Caesarean delivery in group 1 (n=61) (number (%))	Caesarean delivery in group 2 (n=69) (number (%))	p-value
MBL (mL)	733±109.8	798±128.7	<0.0001

The difference in the two groups, as reflected in the fall of haemoglobin levels from before to after delivery, is statistically significant (p<0.0001). The drop in Hb levels was less in group 1 as compared to group 2 (Table 6).

**Table 6:** Difference in haemoglobin levels before and after delivery (n=142) Group 1: carbetocin, group 2: oxytocin Hb: haemoglobin level, SD: standard deviation

Parameter	Group 1 (n=142) (mean±SD)	Group 2 (n=142) (mean±SD)	p-value
Hb (gm/dL)			
Pre-delivery Hb	10.6±1.26	10.7±1.19	0.432
Post-delivery Hb	9.43±1.27	9.34±1.22	0.084
Hb difference	1.17±0.19	1.36±0.21	0.0001

The incidence of side effects was significantly lower in group 1 as compared to group 2 (p=0.019). In group 1, 7.8% of participants experienced side effects compared to 16.9% of participants in group 2. The most common side effect observed in both groups was flushing, which was seen in five (3.5%) women in group 1 and 12 (8.5%) women in group 2. Vomiting was reported in two (1.4%) women in group 1.

## Discussion

The demographics show no statistically significant variation between the groups and thereby eliminate the demographic confounding factors.

The rates of PPH in the present study did not differ significantly in the groups receiving prophylactic carbetocin or oxytocin, irrespective of the mode of delivery; however, there was a significant difference between the two groups in the amount of mean blood loss and haemoglobin decline (pre- and post-delivery) being less in the carbetocin group when compared to the oxytocin group.

The rate of PPH as the primary outcome of the present study is in accordance with the CHAMPION trial by Widmer et al., who reported that carbetocin was non-inferior to oxytocin in preventing blood loss of 500 mL or more, with rates of 14.5% and 14.4%, respectively, demonstrating that carbetocin effectively prevents moderate blood loss during vaginal delivery [10]. Further, the Cochrane Database of Systematic Reviews (2025) concluded with high certainty evidence that carbetocin has a similar effect compared with oxytocin for preventing PPH of 500 or 1000 mL [9], and Jin et al., in their large-scale retrospective analysis, found no significant difference in the rate of PPH ≥ 500 mL or PPH ≥ 1000 mL between carbetocin and oxytocin [14]. Another meta-analysis by Jin et al. also supports our results by concluding that carbetocin was as effective and safe as oxytocin for the prevention of postpartum haemorrhage in women undergoing vaginal delivery [15].

Few studies have shown that carbetocin reduced the incidence of PPH ≥ 500 mL in comparison to oxytocin. Korb et al. reported PPH in 4% versus 5.8% (p=0.004); however, no difference was found with oxytocin in preventing severe atonic PPH after vaginal delivery [16]. Tai et al. observed a significant statistical difference in the incidence of PPH in the carbetocin and oxytocin groups (11% versus 23%; p=0.037) after vaginal delivery; the mode of administration of carbetocin in their study was intravenous, whereas oxytocin was given intramuscularly [17]. It is seen that at many facilities in India, oxytocin is usually kept at room temperature. Even if kept in the fridge, a 24-hour supply of electricity cannot be guaranteed. This barrier of maintaining optimal temperature leads to degradation and loss of efficacy, and thereby suboptimal efficacy, and may account for even better results observed with carbetocin.

In the present study, there was a statistically significant difference in the mean amount of blood loss in the two groups (p=0.039), being less in those receiving prophylactic carbetocin, particularly in women who delivered by caesarean section. This finding is in accordance with other researchers, such as Trivedi et al. (p=0.006) [18] and Rao et al. (p=0.04) [19]. The study done by Tai et al. observed that the mean blood loss was less in the carbetocin group; however, the difference was not statistically significant (p=0.464) [17]. Mohamed et al. found that the blood loss in the oxytocin group was significantly higher than that in the carbetocin group, but not to the extent of PPH [20].

Thus, carbetocin may be more effective in reducing overall blood loss during the third stage of labour compared to oxytocin, highlighting its potential advantage as a uterotonic agent in the prevention of postpartum haemorrhage. The overall significant reduction in the mean blood loss in the carbetocin group of our study may be explained by the fact that although oxytocin was available in our hospital, it was kept in the fridge after being procured, but we could not ascertain the maintenance of the cold chain before procurement by the hospital.

Even if the incidence of PPH remains comparable in the two groups, the reduced amount of mean blood loss may be immensely beneficial to the already anaemic women of LMICs, where saving every drop of blood is crucial for their health.

On evaluating the side effect profile of both drugs, carbetocin was found to have significantly fewer side effects than oxytocin. This is in accordance with the results of the meta-analysis conducted by Ai et al., which included 17 randomised controlled trials (RCTs) involving 32,702 women. About 24 side effects were reported, with a lower risk of vomiting with carbetocin, but no difference among other side effects [21]. Widmer et al. found that the percentages of women who had at least one serious adverse event were similar in both groups, with no significant differences between the two groups [10]. In the study by Tai et al., the adverse effects of drugs were reported to be almost similar in both groups [17].

Our study showed that the haemoglobin decline was less in the carbetocin group, with 1.17 gm/dL, compared to the oxytocin group, with 1.36 gm/dL, and the difference was statistically significant (p=0.002); this is in agreement with the study by Rao et al. [19]. In contrast, Vernekar et al. showed a contrary result, but with a very small and insignificant difference (1.2 g/dL for carbetocin and 1.1 g/dL for oxytocin; p=0.0786) [22]. Our study is also in agreement with the very recent meta-analysis conducted by Maged et al. (2025), who concluded that carbetocin administration in women at low risk for PPH is associated with lower haemoglobin drop when compared to those who underwent oxytocin administration without an increase in adverse effects [23].

The latest Federation of Obstetric and Gynecological Societies of India (FOGSI) recommendations released in 2025 using the latest guidelines advocated by FIGO, WHO and FOGSI recommend, “If oxytocin is unavailable or its quality cannot be guaranteed, the use of other injectable uterotonics, including carbetocin (heat stable) 100 microgram IM/IV is recommended for the prevention of PPH” [12].

As this study shows that carbetocin is almost as effective as oxytocin and it has better results in terms of reducing the mean blood loss and haemoglobin decline, this could be significant in areas where the maintenance of strict temperature control required for oxytocin is uncertain or difficult. However, there are a few limitations, such as a modest sample size and being a single-centre and non-randomised study. Thus, further research with a larger sample size is required to establish the effectiveness of carbetocin in the prevention of PPH.

## Conclusions

Pragmatic strategies for the effective prevention of PPH are the need of the hour. The quality of oxytocin remains uncertain because of the unreliable cold chain maintenance, particularly in low-resource settings situated in tropical and temperate regions. The present study is a milestone towards comparing the effectiveness of heat-stable carbetocin with oxytocin in the prevention of PPH at a tertiary care centre in Central India, where the climate fluctuates widely. The study concluded that carbetocin plays an effective role in preventing PPH, although not in significantly greater proportions when compared to oxytocin, but nonetheless, it was associated with less amount of mean blood loss and haemoglobin decline, with a statistically significant difference. The side effects were also found to be less in the carbetocin group.

In countries like India, with a high prevalence of anaemia, every drop of blood counts, and therefore, liberal adoption of heat-stable carbetocin as a standard practice can be a crucial step to achieve maternal health goals and increase the efficiency of the public health system, where inconsistent temperature control system for oxytocin transport and storage is a barrier for the optimal potency of the drug.
